# Cellular scars and local crosstalk in relapsing psoriasis: an example of a skin sticking disease

**DOI:** 10.1111/sji.12953

**Published:** 2020-09-19

**Authors:** Irène Gallais Sérézal, Stanley Cheuk, Elisa Martini, Liv Eidsmo

**Affiliations:** ^1^ Department of Medicine Unit of Rheumatology Karolinska Institutet Solna Stockholm Sweden; ^2^ Department of Dermatology Besançon University Hospital Besançon France; ^3^ Department of Rheumatology and Inflammation Research University of Gothenburg Gothenburg Sweden; ^4^ Department of Paediatrics University of Oxford Oxford UK; ^5^ Diagnostiskt Centrum Hud Stockholm Sweden

**Keywords:** T Cells, cells, autoimmunity, diseases, chemokines, molecules, inflammation, processes, memory, psoriasis, human, subject, skin, tissues

## Abstract

Psoriasis is an inflammatory disease that arises in genetically predisposed individuals. Chronic skin lesions that contain activated immune cells can persist for years. Systemic inhibition of TNF, IL‐17 and IL‐23 cytokines has revolutionized psoriasis care during the recent decades. Unfortunately, local relapse of disease is common at previously inflamed sites after cessation of treatment. This highlights that fundamental pathologic alterations of the affected tissues are not completely resolved during clinical remission. Here, we present arguments for a local disease memory located in both dermis and epidermis in psoriasis skin. We decipher different cellular components and intercellular crosstalk that sustain local disease memory and amplify disease relapse in human psoriasis. Decrypting the mechanisms underlying the establishment and persistence of pathogenic memory cells in resolved psoriasis may provide new therapeutic perspectives aimed at long‐term remission of psoriasis.

## 1 INTRODUCTION

Psoriasis is a common disease primarily associated with red and scaly patches on the skin. Arthritis affects one‐third of the patients, and systemic disease occurs in severe cases with the alteration of circulating immune cells and cytokines.^1^, ^2^ However, the majority of patients develop mild disease with a few lesions. Local recurrence of disease is common in areas of the skin previously affected by psoriasis. The existence of a local memory would explain the local recurrence of disease in fixed patches of skin, opening up for local therapies aimed at preventing the disease from coming back in previously affected areas. Psoriasis arises in genetically predisposed subjects following environmental triggers.^3^ Genome‐wide linkage analyses have repeatedly shown the association of MHC class I loci with psoriasis and HLA‐Cw6 located in the psoriasis susceptibility locus 1 (PSORS1) accounts for up to half of disease heritability.^4^, ^5^ This indicates aberrant crosstalk between skin stromal cells and T cells. A number of antigens have been proposed to drive psoriasis, including the antimicrobial peptide LL‐37, the melanocyte‐antigen ADAMTSL5, keratinocyte‐derived keratins and streptococcal antigens.^6^, ^7^, ^8^, ^9^, ^10^

**Figure 1:**
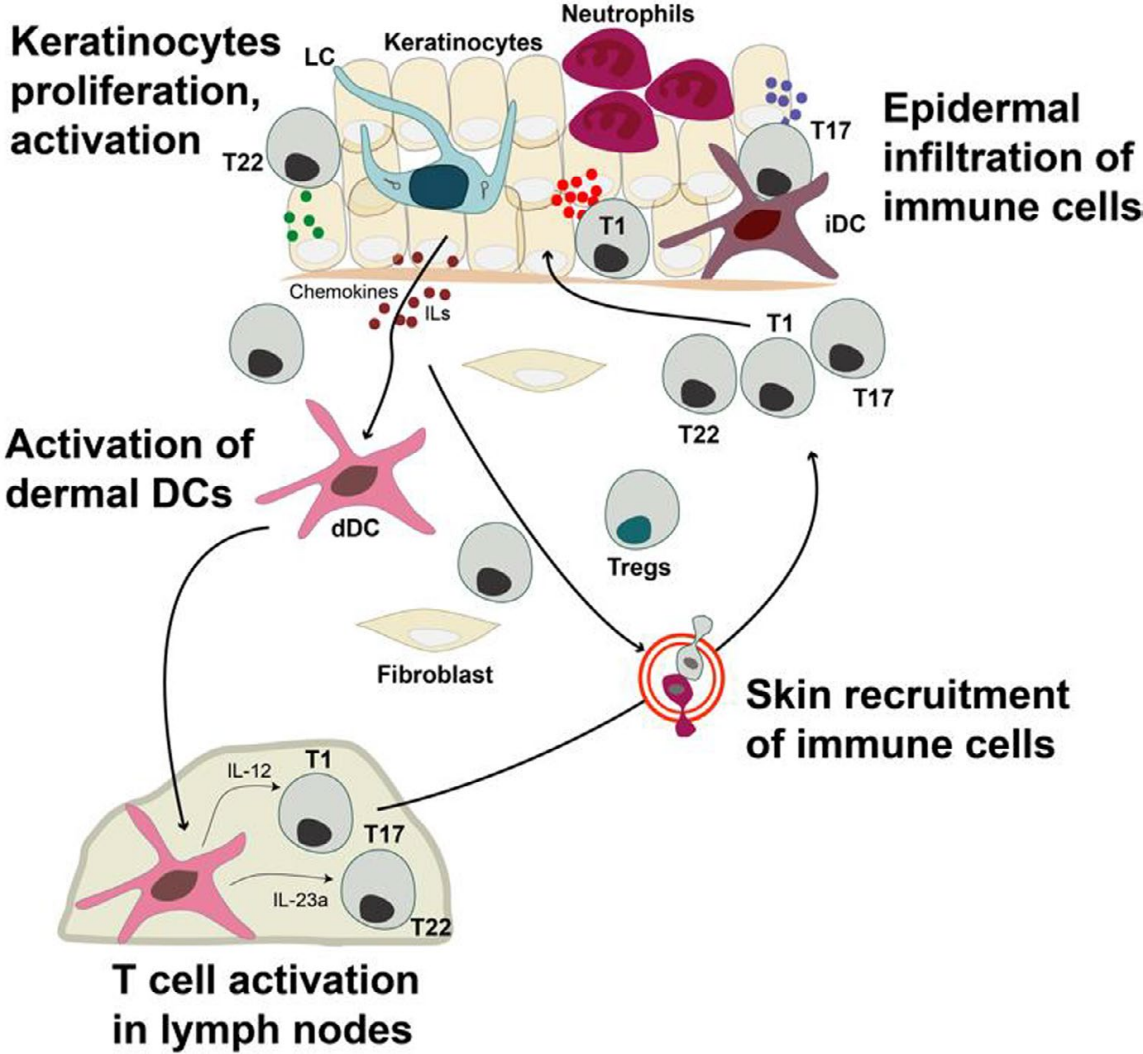
Immune deviations in active psoriasis lesions. In active psoriasis lesions, the non‐resolving inflammation stems in intercellular pathogenic crosstalks. Migratory and resident immune cells, stromal cells, take part in this process and communicate via chemokines and cytokines. iDC: infiltrating epidermal DC; dDC: dermal DC; LC: Langerhans cell; T17: T cells producing IL‐17; T1: T cells producing IFNG; T22: T cells producing IL‐22; ILs: interleukins

Here, we focus on established psoriasis lesions, and the skin after the inflammation has resolved. During active disease, massive infiltration by several types of immune cells is obvious in both dermis and epidermis of chronic lesions, including IFN‐γ, IL‐17 and IL‐22 producing T cells, inflammatory IL‐23 producing dendritic cells, neutrophils and monocytes/macrophages (Figure 1). In the dermis, the infiltrated cells predominate around dilated blood vessels, and the epidermis becomes thicker, with altered keratinocytes’ maturation and an increased production of chemokines and antimicrobial peptides such as CCL‐20 and Beta2 defensins in response to the surrounding pro‐inflammatory cytokines.^11^, ^12^

After successful treatment, scarring is unapparent and the macroscopic pathology is completely normalized. However, discreet differences persist in the local immune cell populations compared to non‐lesional skin^13^, ^14^ and clear differences at the molecular level are observed in resolved psoriasis. In 2010, the Krueger laboratory pioneered the field when Suarez‐Farinas et al showed dysregulated mRNA expression in full‐thickness skin biopsies of resolved psoriasis after three months treatment with systemic TNF inhibition compared to non‐lesional skin.^15^ Pathway analysis showed that this 'molecular scar' of transcripts in resolved skin was linked to cytotoxic T cells and that the level of expression of transcripts such as *GZMA* and T cell receptor β‐constant 1 (TRBC1) were less than 75% normalized. An altered transcriptional signal can be explained by a quantitative imbalance of cell populations that are not back to prelesional levels. Additionally, post‐transcriptional and epigenetic changes can also participate in the gene expression modification in the tissue. Hence, in 2012, Roberson et al showed that DNA methylation pattern only modestly differed between lesional skin and resolving skin after one‐month treatment with TNF blockers, indicating that epigenetic changes could participate in the molecular scarring process.^16^ Another study investigated microRNA expression levels in treated skin with TNF blockers and found that miR31 levels were still increased in skin after 80 days of treatment. MiR31 is pro‐inflammatory in psoriasis skin by regulating the production of inflammatory mediators, modulating leucocyte chemotaxis to the skin and promoting hyperplasia.^17^, ^18^ These studies suggest that macroscopic status does not reflect the molecular state of the resolved psoriasis. Here, we review mechanisms and cell types that may participate in the disease memory and in the local relapse.

## 2 THE CELLULAR COMPONENTS OF LOCAL MEMORIES IN THE SKIN

### 2.1 T cells and Tissue‐resident memory cells

The pathogenic role of T cells in psoriasis has been shown in different settings. Already in the 1990s, several attempts to treat psoriasis through systemic infusion of monoclonal antibodies depleting CD3^+^ or CD4^+^ T cells showed reduced severity of psoriasis in patients.^19^, ^20^, ^21^ Professor Nickoloff proved the role for T cells in psoriasis pathogenesis using a xenotransplantation model, where human skin was transplanted onto immunocompromised mice (SCID). The maintenance of skin pathology within grafted lesional psoriasis skin was shown not only to be T cell‐dependent but skin‐derived T cells were more efficient in maintaining pathology as compared to blood‐derived T cells from psoriasis patients.^22^ Follow‐up studies highlighted that intradermal injection of preactivated blood‐derived activated CD4^+^ T cells could induce active psoriasis in uninvolved skin from psoriasis patients.^23^

Functional disequilibrium between skin and blood T cells were proven in pioneer work from the Carbone laboratory utilizing recall responses to cutaneous herpes simplex virus (HSV) infection in mice. Gebhardt et al showed that HSV‐specific CD8^+^ T cells expressed CD69 and CD103 and preferentially persisted in previously infected skin epithelia where these cells provided local recall response against HSV reinfection in the skin.^24^ This resident population was then termed tissue‐resident memory T (T_RM_) cells, and their protective role in local adaptive immune defences has been further confirmed in other non‐lymphoid tissues and in lymph nodes.^25^, ^26^, ^27^, ^28^ The main focus was long on CD8^+^ T_RM_ cells, but CD4^+^ T_RM_ were recently shown to be important as well in antimicrobial defence.^29^ Additionally, they seem more prone to recirculation from their non‐lymphoid organ to the blood circulation than the CD8^+^ counterparts.^30^ How long these cells can persist in the skin is unknown, but in human fixed drug eruption, pathogenic epithelial CD8^+^ T cells were shown to persist for years.^31^ Initial evidence for pathogenic T_RM_ cells in psoriasis was presented in another xenotransplantation model where uninvolved skin from psoriasis patients was transplanted onto severely immunocompromised (AGR) mice. In this model, psoriasis spontaneously developed in the absence of blood circulation. Depleting T cells prevented disease, which implicated that psoriasis development is T_RM_ cell‐dependent.^32^ Subsequently, epidermal infiltration of CD49a–bearing T cells was associated with the development of psoriatic inflammation,^33^ stressing the importance of the epidermal compartment in local development of the disease.

Already in 1985, Baker and colleagues had shown that a decrease of epidermal T cells precedes the clearance of inflammation during UVA treatment in psoriasis.^34^ Furthermore, the efficacy of ablating epidermal T cells correlated with the clinical amelioration of the lesions. We have previously showed that epidermal T cells in resolved psoriasis following UVB treatment, TNF or IL‐12/23 inhibition contained CD8^+^ T_RM_ cells poised to produce IL‐17 and CD4^+^ T_RM_ cells poised to produce IL‐22.^13^ The importance of T_RM_ surface markers for their function was first observed in an elegant paper by the Clark laboratory in which CD69^+^ T cells expressing CD103 produced more IFN‐γ in epidermis, and more IL‐22 and TNF in the dermis, upon ex vivo activation.^35^ We have further showed that IL‐17 expression in epidermal CD8 ^+^ T_RM_ cells in resolved skin was primarily confined within the CD8^+^CD103^+^CD49a^‐^ T_RM_ cell subset highlighting the possibility that CD49a^+^ and CD49a^‐^ CD8^+^ T_RM_ cells have different roles during the active and resolved phases of psoriasis.^36^ The IL‐17 producing T cells in resolved psoriasis were shown by the Clark laboratory to have more clonal overlap with T cells in lesional skin from the same patients, than in the non‐lesional skin, indicating that the putative pathogenic T_RM_ cells in resolved psoriasis were 'leftovers' from the active phase of the inflammation.^37^ Intriguingly, Matos et al identified CDR3 sequences in the skin that were shared among several patients, but absent in healthy skin and in other inflammatory skin diseases. The epidermal and dermal compartments, however, were not analysed separately.

### 2.2 Regulatory T cells

After treatment is stopped, skin homoeostasis is often maintained for months, and sometimes years, despite the presence of IL‐17 producing epidermal T_RM_ cells. The speed of relapse of the psoriasis lesion is highly variable. The environmental influence on clinical manifestations of psoriasis is obvious, and many patients correlate relapse with increased stress, infections or other environmental triggers. On a cellular level, the density of pathogenic T cells remaining in the tissue may play a role, but several studies indicated that homoeostasis is maintained by tolerogenic cells within the tissue. The proportion of regulatory T cells (Tregs) among CD4^+^ T cells is lower in both lesional and non‐lesional skin compared to levels in healthy skin.^38^ Interestingly, Kotb et al also showed that the density of Tregs increases during UVA treatment or topical application of D vitamin and betamethasone.^38^ In contrast, Keijsers et al observed a decrease in Tregs analysed by IHC staining in 12 psoriasis patients treated with topical application of D vitamin and betamethasone, although with a high interindividual variation.^39^ In this latter article, the skin of patients treated with the TNF blocker adalimumab, however, exhibits an increased Foxp3/CD4 ratio after treatment compared to before treatment, in line with Kotb et al. These studies did not present epidermal and dermal data separately but Tregs likely persist in the dermis, as *FOXP3* expression in CD4^+^ T cells from resolved epidermis after both UVB and biologics treatment is not significantly different from healthy skin.^13^ Antiga et al observed a 2‐fold increase in CD4^+^ T cells producing IL‐10 by flow cytometry after 12 weeks of treatment with TNF inhibition.^40^ Unfortunately, it is not known if these changes correlate with treatment efficacy or disease outcome. Finally, it would be intriguing to investigate whether these alterations in dermal CD4 T cells are as long‐lasting as those observed in T_RM_ populations in the epidermis.

### 2.3 Dermal dendritic cells, plasmacytoid dendritic cells

Dendritic cell (DCs) populations, including Langerhans cells (LCs), are innate immune cells that are heavily affected by the inflammatory state of the skin. During steady state in healthy skin, dermal DCs (dDCs) reside and surveil the environment in the dermis.^41^ In the epidermis, LCs use protruding dendrites to constantly sense the presence of antigens penetrating the stratum corneum as well as the local inflammation within the epidermal compartment.^42^ DCs have an important role in establishing and influencing the function of memory T cells in the skin. In recent years, the concept of innate immune memory, also called trained immunity, has been proposed to explain observations of the ability of vertebrates to mount more powerful innate immune response to repeated encounters.^43^ In contrast with 'classical' immune memory, innate immune memory is short‐lived and relies on epigenetic changes.^44^ Whether skin DCs can be considered as part of innate immune memory cells remains to be shown.

The local pool of DCs is profoundly re‐shaped during inflammation in psoriatic skin. The number of DCs in the dermis increases drastically, and Langerin^‐^ DCs are observed in the epidermis.^45^, ^46^, ^47^ Initial studies to define dDCs in psoriatic lesions used the marker CD11c,^48^ which does not distinguish resident vs infiltrated populations. In active lesions, dDCs display morphological changes and a pro‐inflammatory profile with an increase in the expression of IL‐12/IL‐23p40 first described by Lee et al^49^ Additionally, TNF– and iNOS–producing dDCs have been identified by independent studies ^45^, ^50^ and we have shown that dDCs produce an array of pro‐inflammatory cytokines including IL‐23 and IL‐1β but also the regulatory cytokine IL‐10.^14^ Plasmacytoid DCs (pDCs) have been implicated in the early stages of psoriasis plaque formation, but their role in sustaining established plaques is not clear, as pDCs swiftly disappear from the skin upon treatment.^51^, ^52^, ^53^

Successful therapies seem to reduce the density of DCs in both epidermis and dermis. A limited amount of studies has explored the functional properties of DCs in resolved lesions. These studies show that the DCs retain the capacity to produce IL‐23 and TNF,^45^, ^54^, ^55^ indicating that they are part of the pool of inflammatory cell persisting locally in clinically resolved disease. Studies on anti‐IL‐17 agents indicated a reduction in the expression of pro‐inflammatory molecules in whole skin specimens, including the DC‐ and macrophage‐specific cytokine *IL23*, after treatment.^56^ On the contrary, the dDCs that infiltrate the epidermis during active psoriasis are absent in resolved epidermis after UVB or during anti‐TNF treatment, indicating a more strict correlation between the presence of infiltrating DCs (iDCs) in epidermis and the clinical disease.^14^

### 2.4 Langerhans cells

The pro‐inflammatory role of epidermal LCs was first inferred by the observation that higher expression of *CXCL1*, *CXCL10*, *CCL18* and *CCL20* was measured in LCs obtained from lesional psoriasis.^57^ Moreover, functional studies demonstrate that LCs can produce IL‐23 after different types of toll‐like receptor stimulation.^14^, ^58^ The production of pro‐inflammatory mediators in connection with the reduced mobility of LCs observed in lesional skin ^59^, ^60^ indicates that LCs are capable of in situ stimulation of epidermal T cells in psoriatic lesions.

We have shown that LCs obtained from the epidermis of clinically healed skin colocalize with epidermal T cells and retain abnormal gene expression profiles.^14^ Of particular interest, *IL23* and *IL15* expression levels are abnormally upregulated in LCs after anti‐TNF treatment, whereas *CCR2* is downregulated. The cytokine IL‐15 and IL‐23 are respectively important for T cell proliferation and the generation of IL‐17–producing T cells, whereas CCR2 marks monocyte‐derived LCs.^61^ In LCs from UVB‐treated epidermis, *IL15* and *CCR2* are upregulated while *IL23* is downregulated, whereas *IL23* is upregulated in anti‐TNF‐treated epidermis.^14^ Thus, even the epidermal compartment retains antigen‐presenting cells that are poised to express pro‐inflammatory genes.

Whether inflammatory LCs and DCs remaining respectively in resolved psoriasis epidermis and dermis can activate or potentiate T cell functions has not been demonstrated yet, but taken the altered gene expression profile of LCs, coupled with their spatial proximity to T cells in the epidermis, LCs may drive in situ T cell activation and local relapse in psoriasis.

## 3 PATHOGENIC CROSSTALKS IN RESOLVED PSORIASIS

### 3.1 DCs as local modulators of T cells pathogenicity?

The T cell activation capacities of DCs and LCs have been extensively studied in murine models, whereas data from human antigen presentation are obtained from in vitro experiments. During steady state, dDCs might be crucial for the generation of skin memory T cells. According to a recent study, skin DCs migrate to the lymph nodes, where they precondition naïve T cells to become 'future' memory T cells by producing TGF‐β.^62^ This priming step was fundamental for effective T cells establishment and persistence in the skin following cutaneous infections. In both human and mouse models, changes in skin homoeostasis lead to the infiltration of additional populations of DCs in the skin. Resident and infiltrated DCs are phenotypically indistinguishable, and both subsets are capable of producing inflammatory mediators like iNOS, TNF and pro‐inflammatory interleukins.^42^, ^63^ In addition to the pro‐inflammatory features, DCs within tissues can also be tolerogenic and DCs form regulatory T cells in the gut.^64^, ^65^ Less is known regarding tolerogenic dDCs within the skin, but the Nestle laboratory and the de Jong laboratory showed, in two different experimental settings, that dDCs are capable of producing IL‐10 which, in turn, can induce tolerogenic features in co‐cultured T cells.^66^, ^67^ The tolerogenic feature of DCs could be advantageous in psoriasis to dampen the inflammatory state in active psoriasis and to prolong the time between relapses in resolved skin.

LCs may interact differently with T cells compared to dDCs. Human LCs are characterized by high expression of CD1a, a membrane receptor specialized in presenting lipid antigens to CD4 T cells. Kim et al showed that the interaction between the antigen urushiol and CD1a‐expressing LCs caused inflammation and IL‐17 production by CD4^+^ T cells in a transgenic mouse model where mouse LCs were engineered to express human CD1a.^68^ Of note, CD1a‐reactive T cells capable of producing IL‐17 and IL‐22 were identified in the blood and skin ^69^ of psoriasis patients, indicating a potential role for lipid antigens in psoriasis.^68^ Pro‐inflammatory LCs that activate T cells in psoriasis are in agreement with persistent *IL23* expression in LCs from resolved psoriasis, and contrast with other skin diseases like allergic contact dermatitis where LCs are more tolerogenic than their dermal DCs counterparts.^70^ Vitamin D3 could modify this balance, as CD25^hi^CD127^lo^FOXP3^+^ inducible Tregs can be obtained after LCs are primed with vitamin D are co‐cultured with allogenic naive T cells, while a similar protocol using dermal DCs instead of LCs led to the development of an IL‐10^+^FOXP3^‐^ T cell subset.^67^ Rácz et al showed that genes belonging to the vitamin D pathway are modulated by UVB therapy in psoriasis,^71^ and others witnessed increased levels of circulating vitamin D during UVB treatment,^72^ but more research is needed to explore whether there is any link between UVB therapy and tolerogenesis mediated by vitamin D‐primed DCs.

### 3.2 Keratinocytes and cytokines: a miscommunication?

The transcriptomic signature in keratinocytes within psoriasis plaques is dominated by the IL‐22 and IL‐17 signalling pathways.^73^ IL‐17 is produced by a number of immune cells within the plaques, including Th17 and Tc17 cells as well as neutrophils, mast cells, and innate lymphoid cells.^74^, ^75^, ^76^, ^77^ IL‐17A is a potent inducer of pro‐inflammatory cytokines and chemokines in stromal cells, especially CXCL1, IL‐8 and CCL‐20, as well as antimicrobial peptides.^78^ IL‐22 is produced by CD4^+^ and CD8^+^ T cells, either alone or in combination with IFN‐γ or IL‐17.^79^, ^80^ In the skin, IL‐22 is a key cytokine for maintaining epithelial homoeostasis and inflammation, and it induces strong antimicrobial responses and proliferation of keratinocytes.^81^ IL‐22 also potentiates the inflammatory response mediated by TNF.^82^ IFN‐γ is produced by resident memory T cells in response to viral infection and induces localized inflammation and antiviral responses.^83^, ^84^ In stark contrast with IL‐22, IFN‐γ inhibits the proliferation of keratinocytes ^85^ and instead induces the production of cytokines (IL‐1, IL‐6 IL‐15), chemokines (IL‐8, CCL‐5, CXCL‐9, CXCL‐10 and CXCL‐11) and upregulation of adhesion molecules.^86^, ^87^ Inflammatory and tolerogenic immune cell‐derived cytokines are secreted simultaneous within the skin, and it is virtually impossible to detangle the effect of individual cytokines from snapshot samples collected from diseased skin.

Furthermore, keratinocytes from psoriasis patients differ from keratinocytes in the skin of healthy individuals. In addition to different patterns of MHC class I expression, psoriasis patients display distinct gene expression signatures and DNA methylation profiles in non‐lesional skin.^88^, ^89^ Keratinocytes from non‐lesional psoriasis skin also exhibit functional differences with decreased triggering of IRF‐1 and STAT1 following exposure to IFN‐γ.^90^ In line with decreased IFN‐γ responses, supernatants from activated lesional T cells led to increased proliferation ^91^ and resistance against UV‐induced apoptosis than healthy counterparts. Taken the localized disease memories created by T cells and LCs in resolved lesions, thorough transcriptomic and epigenetic characterization of keratinocytes from resolved epidermis could further reveal mechanisms of relapsing psoriasis. Keratinocytes collected from resolved imiquimod‐induced dermatitis in mice showed profound epigenetic changes and lower threshold for inflammasome activation.^92^ Whether similar mechanisms are present in resolved psoriasis in humans is yet to be determined.

### 3.3 The skin as a local amplifier of T cell pathogenicity?

Work from several laboratories, including ours, has highlighted retention of pathogenic T_RM_ cells in resolved psoriasis,^13^, ^37^, ^93^ suggesting that T_RM_ could dictate local relapse. Taken the multitude of responses that are induced by cytokines simultaneous produced in three dimensional tissues in vivo, it is difficult to model how—and if—T_RM_ cells drive local relapse. More importantly, it is puzzling that cells capable of producing IL‐17 in resolved lesions do not cause pathology in most resolved lesions. Keratinocytes from patients with psoriasis differ genetically from available cell lines, and two‐dimensional keratinocyte cultures react differently than stratified epithelia. To characterize tissue responses to T_RM_ activation in resolved skin, we activated T_RM_ cells ex vivo in skin biopsies using anti‐CD3 antibody (OKT‐3). T_RM_ activation induced IL‐17 and interferon signals in the resolved epidermis. Type I interferons signalling, a known trigger of human psoriasis,^94^ was induced by T_RM_ cell activation or IFN‐γ signalling.^93^ Importantly, the balance of IFN‐γ, IL‐10 and IL‐17 induced T_RM_‐driven responses correlated with time in remission following UVB treatment, which suggests that the balance of different subsets of T_RM_ cells is critical for long‐term control of psoriasis lesions. These data indicate that the combined effect of LC and T cell‐derived cytokines on keratinocytes reflect the depth of the disease 'dormancy' during triggering events. The concept of T_RM_ cell balance to promote long‐term resolution is currently tested in a randomized clinical trial of early‐onset psoriasis.^95^

## 4 CONCLUSION

**Figure 2:**
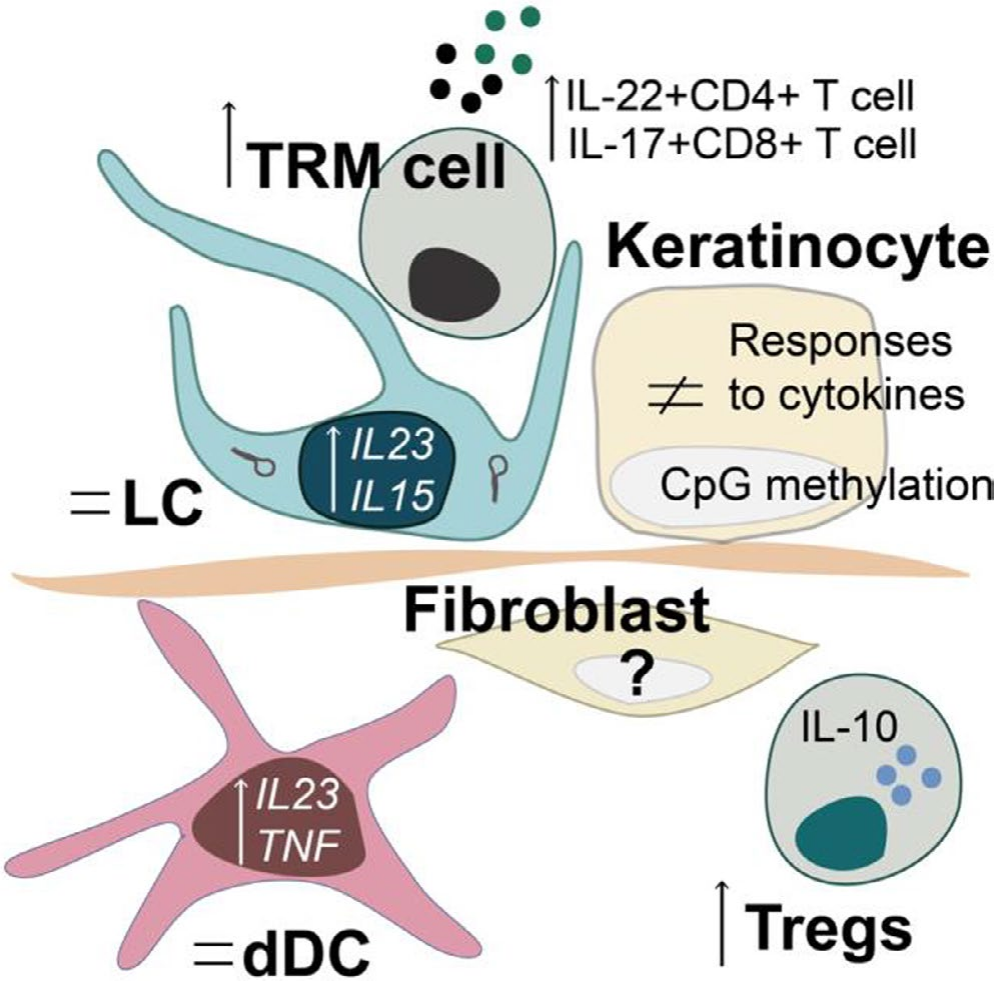
Cellular and molecular participants of the disease memory in psoriasis. In resolved skin, T_RM_ cells and keratinocytes retain pro‐inflammatory abilities with respectively an increased capacity to produce psoriasis‐relevant cytokines and an altered response to the cytokines. In LCs and dDCs, pro‐inflammatory transcriptional changes are observed, while Tregs are increased. Thorough characterization of the fibroblastic changes is missing. '='refers to the stability in cell number or proportion, and arrows represent increases (up) or decreases (down) in the corresponding cell type or RNA expression. dDC: dermal DC; LC: Langerhans cell; T_RM_ cell: tissue‐resident memory T cell; Tregs: regulatory T cells; IL: interleukin

Psoriasis is a patchy skin disease that resolved without scarring but often relapses in previously affected sites. Despite the improvement of care through a smorgasbord of novel systemic treatments for severe psoriasis, topical corticosteroids with vitamin D treatments and light treatment are the only available therapies for mild diseases. Mechanisms of recurrent skin pathology would be an attractive target for novel topical treatments of this common disease. We and others have shown that pathogenic T_RM_ cells poised to IL‐17 production persist in close vicinity to LCs with elevated expression of *IL23* or *IL15* and surrounded by keratinocytes in the epidermis of resolved psoriasis (Figure 2). In favour of T_RM_‐driven recurrent pathology, the strength of IL‐17 signalling in epidermis following T_RM_ cells activation ex vivo correlates to time in remission. However, causality is not fully established and future studies with prospective follow‐up of T_RM_ function during the disease treatment and relapse are necessary to prove that T_RM_ cells are truly pathogenic in resolved psoriasis. Finally, targeting T_RM_ cell survival in resolved tissue would attest the role of T_RM_ cells in psoriasis pathogenesis.

## CONFLICT OF INTEREST

LE has received consultant fees from Novartis and Leo Foundation.

## AUTHOR CONTRIBUTIONS

IGS designed the first outline of this review. IGS, SC and EM reviewed the outline and wrote the first draft of the manuscript. LE revised the manuscript, and all authors reviewed the final manuscript.
